# Profiling risk factors for chronic uveitis in juvenile idiopathic arthritis: a new model for EHR-based research

**DOI:** 10.1186/1546-0096-11-45

**Published:** 2013-12-03

**Authors:** Tyler S Cole, Jennifer Frankovich, Srinivasan Iyer, Paea LePendu, Anna Bauer-Mehren, Nigam H Shah

**Affiliations:** 1 Stanford Center for Biomedical Informatics Research, Stanford University School of Medicine, 1265 Welch Road, MSOB, X-215, Stanford, CA 94305-5479, USA; 2 Division of Rheumatology, Department of Pediatrics, Stanford University School of Medicine, Palo Alto, CA, USA

**Keywords:** Juvenile idiopathic arthritis, Uveitis, Allergy, Electronic health records, Text mining, Biomedical informatics

## Abstract

**Background:**

Juvenile idiopathic arthritis is the most common rheumatic disease in children. Chronic uveitis is a common and serious comorbid condition of juvenile idiopathic arthritis, with insidious presentation and potential to cause blindness. Knowledge of clinical associations will improve risk stratification. Based on clinical observation, we hypothesized that allergic conditions are associated with chronic uveitis in juvenile idiopathic arthritis patients.

**Methods:**

This study is a retrospective cohort study using Stanford’s clinical data warehouse containing data from Lucile Packard Children’s Hospital from 2000–2011 to analyze patient characteristics associated with chronic uveitis in a large juvenile idiopathic arthritis cohort. Clinical notes in patients under 16 years of age were processed via a validated text analytics pipeline. Bivariate-associated variables were used in a multivariate logistic regression adjusted for age, gender, and race. Previously reported associations were evaluated to validate our methods. The main outcome measure was presence of terms indicating allergy or allergy medications use overrepresented in juvenile idiopathic arthritis patients with chronic uveitis. Residual text features were then used in unsupervised hierarchical clustering to compare clinical text similarity between patients with and without uveitis.

**Results:**

Previously reported associations with uveitis in juvenile idiopathic arthritis patients (earlier age at arthritis diagnosis, oligoarticular-onset disease, antinuclear antibody status, history of psoriasis) were reproduced in our study. Use of allergy medications and terms describing allergic conditions were independently associated with chronic uveitis. The association with allergy drugs when adjusted for known associations remained significant (OR 2.54, 95% CI 1.22–5.4).

**Conclusions:**

This study shows the potential of using a validated text analytics pipeline on clinical data warehouses to examine practice-based evidence for evaluating hypotheses formed during patient care. Our study reproduces four known associations with uveitis development in juvenile idiopathic arthritis patients, and reports a new association between allergic conditions and chronic uveitis in juvenile idiopathic arthritis patients.

## Background

Juvenile idiopathic arthritis (JIA) is the most common rheumatic disease in children, with prevalence rates similar to juvenile-onset diabetes, as high as 4.01 per 1,000 children [1]. Chronic uveitis is the most threatening co-morbid condition seen in JIA patients and affects between 2% and 38% of children with arthritis [2]. Untreated uveitis can lead to cataracts, glaucoma, band keratopathy, retinal detachment and vision loss [3]. Most JIA patients with uveitis have asymptomatic eye disease [4] and, due to their young age, are unable to articulate and/or recognize the vision changes; because of this, clinicians must screen for uveitis routinely.

Current screening guidelines are based on the understanding of two risk factors, age and ANA status [5]. Such algorithms have been the backbone of curtailing ocular complications of uveitis [2], and the discovery of novel associations will improve risk stratification with regular screening. The knowledge embedded in clinical documents from electronic health records—used, for example, to inform therapy decisions in juvenile systemic lupus erythematosus [6]—could enable such discovery for JIA and uveitis.

With computational advances in processing unstructured clinical data, large repositories of clinical data have been used for pharmacovigilance [7], phenotypic profiling [8], and for generating practice-based evidence [9]. With structured billing and claims data complemented by the rich content of clinical text, researchers argue that much of clinical medicine can benefit from analyzing data already in clinical data warehouses [6,7,10-17]. Investigators can use this data to reveal associations and predictors for hard to detect, yet severe, disease complications and co-morbidities.

Based on clinical observations, we hypothesized that allergic conditions may be associated with uveitis in JIA patients and examined this association via an informatics approach. We tested for allergy associations by mining unstructured clinical notes and coded data. Although the methods applied have been validated in other studies [7,9,18-21], as an internal validation we reproduced previously reported associations of uveitis including age [22-26], oligoarticular-onset disease [3,22-25,27], antinuclear antibody (ANA) status [22-25,27], rheumatoid factor (RF) status [22,23,28], and the presence of psoriasis in the patient or in immediate relatives [29]. This study adds to a growing literature demonstrating the potential of analyzing clinical data warehouses for rapidly evaluating a clinically formed hypotheses using practice-based evidence [11,30].

## Methods

### Data source

Our patient population was drawn from the Stanford Translational Research Integrated Database Environment (STRIDE), containing data from 1.8 million patients from the Stanford Hospital and Clinics and the Lucile Packard Children’s Hospital. Acquisition and processing of data was approved by the Stanford Institutional Review Board.

### Patient population

Patients followed by the pediatric rheumatology division at Lucile Packard Children’s Hospital were included in the study. The pediatric rheumatologists at our center participate in the medical management of uveitis in conjunction with a community and/or university ophthalmologist. Each clinical encounter is coded with diagnosis codes for JIA and uveitis (if present) using a standardized rheumatology clinic billing form. ICD-9 code standards have not changed during the study time period. Summaries of all clinical patient encounters are dictated by pediatric residents, rheumatology fellows, and pediatric rheumatology attending physicians using our clinic encounter template form which includes medication reconciliation of both prescription and non-prescription medications.

### Cohort selection

**Figure 1:**
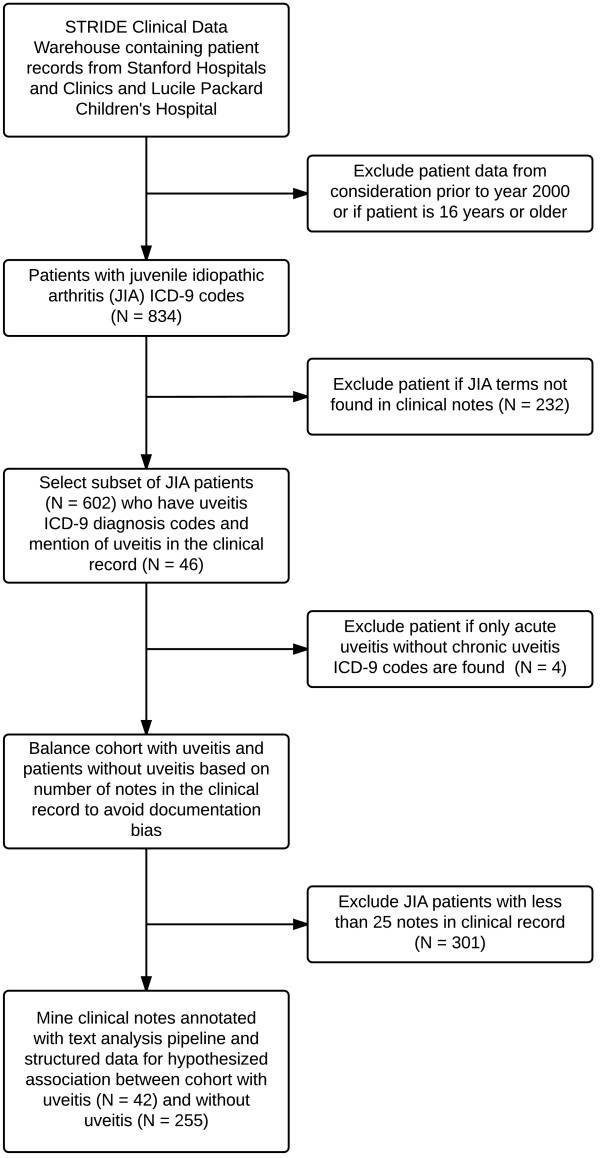
**Cohort selection process using both structured ICD-9 codes and unstructed notes annotated with text analysis.** From 602 JIA patients, 42 chronic uveitis patient were selected with ICD-9 codes confirmed with mention of uveitis terms in their clinical notes. JIA patients with under 25 notes in the clinical database were excluded to prevent documentation bias.

Cohort selection was based on both ICD-9 diagnosis codes and the contents of the processed clinical notes relating to the diagnosis as outlined in Figure 1. A dual-layered method of patient identification was used to avoid the selection of incorrectly coded patients, a common and well-documented error in the medical literature [31-33]. The ICD-9 codes and terms used to identify patients are shown in Table 1. Patients under age 16 with JIA were identified between 1/1/2000 until 12/31/2011 (N = 602) and merged with patients diagnosed with chronic uveitis to obtain a cohort of JIA patients with chronic uveitis and a cohort with JIA alone. Patients with only a diagnosis of acute uveitis (ICD-9 code 364.0) were included; those with no diagnosis of chronic uveitis (ICD-9 code 364.1) were excluded (N = 4). Since patients without uveitis tended to have fewer notes in their clinical records, patients with less than 25 clinical notes were excluded to balance this variable and prevent documentation bias. Cohort characteristics and balance are shown in Table 2.

**Table 1:** ICD-9 codes and terms used in cohort selection and potential predictors of chronic uveitis

**Primary cohort (juvenile idiopathic arthritis)**	Outcome of interest (chronic uveitis)*	**Reported and hypothesized patient factors associated with uveitis**
**Juvenile Arthritis ICD 9 codes**	**Uveitis ICD 9 codes**	**Medical status terms in dictated records:**
696.0, 714.0, 714.2, 714.3, 714.9, 720.2, 720.9	364.00 (acute)*	Ana positive, positive ana, psoriasis, allergic, allergy, oligoarticular, oligo-onset, pauciarticular, pauci-onset, monoarthritis, monoarticular, rheumatoid factor positive, rf positive
	364.10 (chronic)*	
**Terms in dictated reports used to confirm diagnosis of juvenile arthritis:**	**Terms in dictated reports used to confirm the diagnosis of uveitis:**	**Examples of allergy medications dictated in clinical records:**
Juvenile idiopathic arthritis, jia, juvenile rheumatoid arthritis, jra, psoriatic arthritis, juvenile spondyloarthropathy, spondyloarthritis, enthesitis related arthritis, sacroiliitis, reactive arthritisand derivatives	Uveitis, iridocyclitis, iritis, and derivatives	Nasal steroids: Flonase, Nasacort
		Oral Antihistamines: Allegra, Zyrtec,
		Claritin, Clarinex, Benadryl, Xyzal
		Nasal antihistamines: Astelin
		Leukotriene inhibitors: Singulair
		Decongestant: Sudafed

*This is the convention used within our institution to differentiate between acute and chronic uveitis.

**Table 2:** Summary of juvenile idiopathic arthritis (JIA) cohort characteristics

	**Controls**	**Cases**	
	**JIA patients with no chronic uveitis**	**JIA patients with chronic uveitis**	
	***N*** **= 255**	***N*** **= 42**	
**Race, no. (%)**			*P =* 0.203*
Asian	23 (9)	6 (15)	
Black	5 (2)	2 (5)	
Other/NA	54 (21)	5 (12)	
White	173 (68)	29 (68)	
**Gender, no. (%)**			*P* = 0.147*
Female	166 (65)	32 (76)	
Male	89 (35)	10 (24)	
**Age, years**	10.2	7.2	*P <* 0.001†
**Number of clinical encounter dictations**	55.4	60.9	*P* = 0.54†

*Difference in proportions analyzed using the Fisher’s exact test.

†Difference in means analyzed with a Student’s t-test.

### Clinical note processing

Textual notes were processed using an optimized version of the NCBO Annotator [19-21] and 22 clinically relevant ontologies. The ontologies provide a lexicon of clinical terms, from which, using a variety of statistical and manual filters [34-38], we removed ambiguous terms including overly frequent or general terms, and misleading abbreviations. We also flagged negated terms and terms attributed to family history [39], and utilized the family history attribution in evaluation of psoriasis as a risk factor. The processing steps are summarized in Additional file 1: Figure S1. We evaluated our clinical note processing pipeline for accuracy in recognizing disease events using a gold standard corpus [40], which has been manually annotated by two annotators for 16 conditions. Overall, our event identification has 74% sensitivity and 96% specificity. Recognition of drug exposure is done in a similar manner, and an independent study [41] estimated over 84% recall and 84% precision for recognizing drugs.

We then used the terms in Table 1 and simple variants of those terms, as well as matching medication names, to determine patient-term attribution. For each patient and term, we further ordered by the time at which a term occurred in this patient–feature matrix.

### Statistical analysis

The clinical notes of the two cohorts were compared for significant differences in the presence of oligoarticular disease in the first 6 months after diagnosis, ANA status, RF status, psoriasis in patient or first degree family member, allergy drugs, and allergy terms listed in Table 1.

Temporal restriction was put on the presence of diphenhydramine to be strictly prior to the first diagnosis of uveitis in the uveitis cohort to prevent confounding by its use to prevent reactions to biologics used to treat uveitis. We manually reviewed the extracted annotated terms and confirmed the presence of the terms in a non-negated context. Drugs were re-coded into a new variable representing the presence of any allergy drug. A two-tailed disproportionality test (Fisher’s Exact) was used to assess association with chronic uveitis.

All variables were then examined in a multivariate logistic regression model with stepwise variable selection. Age at first arthritis diagnosis was highly associated with uveitis and was included in the logistic regression model for adjustment. Although the cohort showed no statistical difference in race and gender (both P > 0.7), these variables were kept in the model for adjustment.

### Cluster analysis

**Figure 2:**
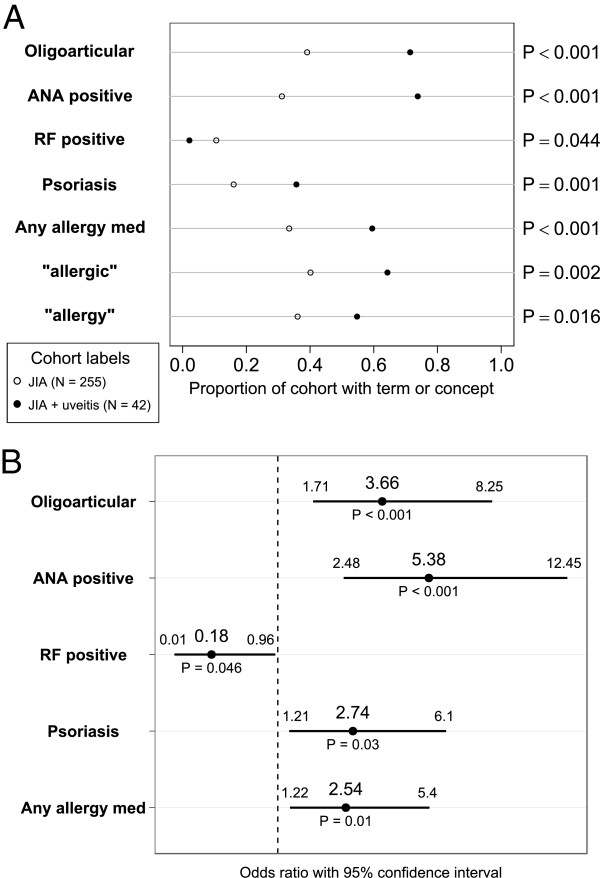
**Patient factors associated with chronic uveitis in juvenile arthritis patients, bivariate analysis and multivariate logistic regression adjusted for age at first arthritis diagnosis, race, and gender. A)** Proportion of cohort with a given term or concept. *P*-values from two-tailed Fisher’s Exact test. **B)** Odds ratios, confidence intervals, and model P values for multivariate association with uveitis.

We then performed unsupervised hierarchical clustering based on the entire patient clinical text content prior to uveitis diagnosis. Terms from the clinical notes of the chronic uveitis patients were limited to the time period prior to the diagnosis of uveitis. The median time offset of uveitis diagnosis from arthritis diagnosis in the uveitis group (282 days) was determined, and used as the maximum time from which to extract notes in the non-uveitis cohort. Terms identified in the clinical notes were converted to medical concepts based on drug and disease ontologies (i.e. mapping similar terms to a shared concept). The presence or absence of each concept was coded into a binary matrix, from which a patient distance (Euclidian) matrix was calculated. Unsupervised hierarchical clustering based on inter-patient distance was then performed. Terms previously used in cohort selection, univariate analysis, or multivariate analysis were excluded from the cluster analysis to understand effects of residual textual features beyond those in Figure 2.

### Software

All statistical analysis was performed using R version 2.15.1 [42].

## Results

### Confirming known risk factors of uveitis

In our data, age at diagnosis in this cohort reflects the age when patients are first seen at our hospital with the diagnosis of JIA. Since some patients transfer their care on referral, the mean age at diagnosis in our cohort is biased towards an older age. The onset of arthritis was earlier in patients who develop uveitis (onset at 7.23 versus 10.17 years, P < 0.001). There was no significant difference in sex between the two groups (P = 0.147), with more females overall.

ANA positivity mentions were increased in chronic uveitis patients (adjusted OR 5.38, 95% CI 2.48–12.45, P < 0.001). Oligoarticular onset pattern was identified as a risk factor (adjusted OR 3.66, 95% CI 1.71–8.25, P < 0.001) based on data from the first 6 months after JIA diagnosis. There was also a positive association between uveitis and psoriasis in the patient or family member (adjusted OR 2.74, 95% CI 1.21–6.1, P = 0.03). An inverse relationship was noted between RF positivity and uveitis (adjusted OR 0.18, 95% CI 0.01–0.96, P = 0.03).

### Testing the hypothesized association between allergies and uveitis

The concept of “allergy” is diffuse, so we examined the hypothesis regarding association of allergy with chronic uveitis in two ways. First, we checked for non-negated mentions of “allergy” and “allergic” within the clinical notes. Second, we created a list and checked for mentions of allergy medications commonly used to treat nasal allergy symptoms in children. We included both over-the-counter and prescription medications, outlined in Table 1.

Allergy drugs were grouped to increase statistical power given the large variety of allergy drugs used and the small patient cohort size. Allergy medication documentation was used as a proxy for nasal allergies; medication reconciliation is a routine part of the medical interview in our pediatric rheumatology clinic and is documented in the clinic visit dictations.

With bivariate analysis, the presence of an allergy drug showed the strongest association with chronic uveitis (OR 2.92, 95% CI 1.47–5.91, P < 0.001). Bivariate analysis also showed the presence of terms “allergic” (OR 2.68, 95% CI 1.34–5.55, P = 0.002) and “allergy” (OR 2.14, 95% CI 1.08–4.27, P = 0.016) to be associated. After finding a positive association with the allergy terms, we subsequently evaluated sinusitis due to its association with allergies. However, terms relating to sinusitis were not statistically associated (OR 1.95, 95% CI 0.94–3.8, P = 0.051).

The variables shown in Figure 2A were incorporated into a multivariate logistic regression model to assess adjusted associations. Age at first arthritis diagnosis was included in the model to account for residual confounding given its strong association. The results of this analysis are shown in Figure 2B. The presence of an allergy drug (OR 2.54, 95% CI 1.22–5.4, P = 0.01) remains associated in the multivariate analysis. There was an overall 14% prevalence of uveitis in our JIA cohort. With estimation of 50% sensitivity for allergy detection and 25% sensitivity of early uveitis detection after referral for continued ophthalmologic evaluation prior to other indications for referral, this would represent a number needed to screen of 95.

The terms “allergy” and “allergic” are independently associated with uveitis (OR 2.14 and 2.68), but less strongly as compared to allergy medications (OR 2.92). Patients who have “allergy” or “allergic” in their clinical notes also tend to have mentions of an allergy drug, therefore the allergy term variable is eliminated in the multivariate model building with stepwise variable selection. When allergy terms are included and allergy medications are excluded in model building, the association with allergy terms remains significant (OR 2.3, 95% CI 1.11–4.96). Manual verification in the 25 chronic uveitis patients with identified allergy drug mentions confirmed direct mentions of allergy conditions in 20 patients. In 3 patients, allergic symptoms and/or consultation with an allergist were directly mentioned. In 2 patients’ clinical text, only direct mention of the allergy drug was identified. Given mandatory medication reconciliation during clinic visits at our institution, and potentially unreliable documentation of allergic conditions such as seasonal allergies due to their high prevalence, mentions of allergy drugs may be a more reliable indicator of allergic conditions. Rheumatologists, ophthalmologists, and pediatricians at our institution do not prescribe the allergy medications identified in our study for general anti-inflammatory purposes.

### Cluster analysis indicating presence of additional associations with uveitis

**Figure 3:**
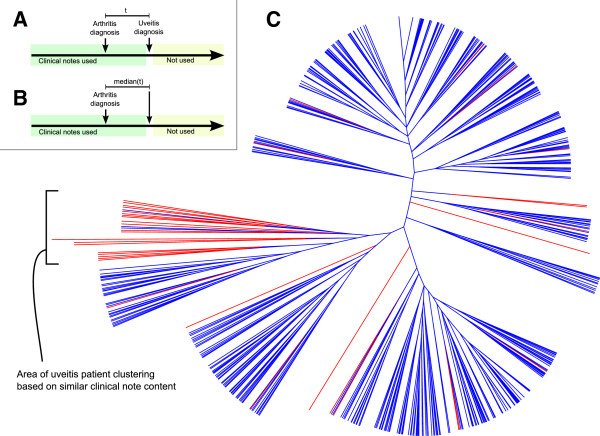
**Phylogram clustering of patients with juvenile arthritis based on residual features in the clinical record prior to uveitis diagnosis. A)** Timeline showing the source of clinical notes in chronic uveitis patients and **B)** non-uveitis patients. **C)** Plot of hierarchical clustering based on the similarity of patient note content. Red represents uveitis patients and blue represents non-uveitis patients.

As a qualitative look into the potential of such analytics on clinical data warehouses to discover additional factors associated with chronic uveitis, we clustered our cohort of patients based on the terms in their clinical record blinded to a future diagnosis of uveitis (Figure 3). In order to investigate if residual associations within the clinical record were strong enough to cluster patients prior to uveitis diagnosis, terms relating to RF status, ANA status, oligoarticular subtype, psoriasis, and allergy were excluded, as were demographic variables and terms already used in cohort selection.

We find that the patients who ultimately develop uveitis do tend to cluster together—even after the exclusion of the strongly associated variables that were the focus of this study—indicating that there are additional predictors within clinical documentation to be investigated. 20 uveitis patients form the main cluster, with only 3 non-uveitis patients included. Influential features of clinical interest include mentions of medications such as beclomethasone, commonly used in pediatric asthma and nasal allergies, and mentions of conditions such as contact dermatitis, folliculitis, and hypersensitivity (P < 0.02).

## Discussion

We examined a hypothesis of a possible association between allergic conditions and uveitis in JIA patients by querying a clinical data warehouse. To validate our methods in the current use case, we demonstrated known positive and negative associations with uveitis in JIA. The onset of arthritis has been demonstrated to be earlier in patients who develop uveitis [26], as shown in the current study. The established associations between uveitis and positive ANA status [22-25], oligoarticular onset pattern of JIA [3,22-25,27], and psoriasis (in patient or first degree family member) [29] is supported by our data. An inverse relationship has been established between RF positivity and uveitis [22,23,28], also confirmed in our study. The ability to also identify negative associations helps to provide an internal validation of the study approach.

After confirming these associations, we present evidence supporting a clinically formed hypothesis about an association between allergic conditions and uveitis. This study highlights the potential of text analytics on unstructured data in clinical data warehouses to examine hypotheses formed during clinical care using practice-based evidence. Without such a computational approach, such hypotheses might be impractical to answer using traditional chart review studies.

We argue that data-mining of electronic medical records—which researchers currently use to inform therapy decisions [6] and enable phase IV surveillance [9,18,19]—should be extended to learn associations and predictors of hard to detect, yet severe, disease complications. Taking such an approach also allows a spectrum of variables to be assessed. Understudied subgroups such as children, the elderly, underrepresented ethnic groups, and pregnant women can be investigated with this approach.

Despite the efficacy of such text-based analyses demonstrated in pharmacovigilance, off-label drug use, and in studying chronic conditions [7,9,18-21], this study has several potential limitations that warrant discussion. Although text analysis techniques achieve 97% accuracy in detecting negated terms, 93% accuracy in detecting drug mentions, and 86% accuracy in recognizing disease conditions in validation studies [7,9,19,20], events occurring outside of the hospital can lead to false negatives. Additionally, it is possible that there is increased reporting of allergic conditions and allergy medications among the chronic uveitis sub-group due to a higher level of concern given the eye disease complication. For these reasons, we feel that a prospective study must validate our findings before allergic conditions can be used as a clinically useful predictor.

In order to account for under-reporting bias arising from fewer visits in patients with less co-morbidity (i.e., the non-uveitis cohort), cases and controls were matched on the number of clinical dictations. The unbalanced JIA cohort without uveitis had approximately half of the number of notes as the cohort with uveitis, and initial analysis including patients with fewer notes revealed stronger positive associations in the uveitis cohort. This was interpreted as falsely strong associations since we could not ensure that patients with less clinical record content were truly negative for a given factor or negative due to less thorough documentation. However, it is possible that patients with fewer notes have less severe disease, biasing the study population to those patients with more severe JIA. This tradeoff to ensure similar medical record information content must be recognized.

Finally, this study does not address causation. Indeed, the association between allergy and uveitis is unanticipated since autoimmune disorders are thought to have a Th_1_/Th_17_ bias while allergic disorders tend to be associated with a Th_2_ cytokine profile. However, this immune classification may be an oversimplification in patients with complex and overlapping diseases, such as those with both uveitis and JIA. Recent investigation suggests that Th_2_ cytokines in the anterior chamber of the eye distinguish patients with idiopathic uveitis from controls using cluster analysis methods [43]. Furthermore, we argue that allergies may be a surrogate risk factor possibly reflecting a heightened immune response to antigens in certain tissues, or a predisposition to both symptomatic and asymptomatic sinusitis where associated bacterial antigens may be the driver of an immune reaction.

If this type of research is performed widely and reliably, then it will become a key aspect of meaningfully using electronic medical records, summarizing practice-based evidence, and can help prioritize prospective clinical trials [6,11,30].

## Conclusion

We report an association between allergic conditions and chronic uveitis in JIA patients. We uncovered this association via analyzing the clinical notes using a validated text analytics data-mining pipeline. If the association is confirmed in prospective studies, it may inform pathogenic mechanisms and help guide clinicians in uveitis screening in patients with allergic conditions. This study shows the potential of analyzing clinical data warehouses to gain new clinical insights and to rapidly prioritize hypotheses formed during clinical care using practice-based evidence.

## Abbreviations

ANA: Anti-nuclear antibody; EHR: Electronic health record; ICD-9: International Classification of Disease version 9; IL: Interleukin; JIA: Juvenile idiopathic arthritis; OTC: Over-the-counter; RF: Rheumatoid factor; STRIDE: Stanford Translational Research Integrated Database Environment; Th: T helper cell.

## Competing interest

The authors declare that they have no competing interest or financial disclosures.

## Authors’ contributions

TSC: Coordination of data collection, study design, data analysis, manuscript drafting and revision. JF: Hypothesis development, clinical correlation, study conception, manuscript drafting and revision. SI: Data collection, clinical text analysis workflow, statistical and computational consulting. PLP: Data collection, clinical text analysis workflow, computational advice, and critical revision of manuscript. AB-M: Statistical consulting, critical revision of manuscript. NHS: Clinical text analysis workflow, methodology for interpreting unstructured EHR data, manuscript drafting and revision, principal investigator. All authors read and approved the final manuscript.

## Supplementary Material

Additional file 1: Figure S1 Generation of the patient–feature matrix. This process (1) starts by downloading ~5.6 M strings for every term in ontologies from both UMLS and BioPortal as well as all trigger terms from NegEx and ConText, (2) uses term frequency and syntactic type information (e.g., predominant noun phrases) from MedLine to prune the set of strings into a clean lexicon, (3) applies the lexicon directly against the textual notes using exact string matching, (4) applies NegEx and ConText rules to identify negated terms and family history contexts respectively, (5) applies UMLS and BioPortal mappings and semantic type information to normalize terms into concepts that are grouped by drug, disease, device, or procedure, (6) and results finally in the patient–feature matrix. Each row of the matrix represents a single patient’s note and the timestamps of the notes induces a temporal ordering over the entire patient–feature matrix. Click here for file
