# Supplementary figures and images for: Profiling risk factors for chronic uveitis in juvenile idiopathic arthritis: a new model for EHR-based research
Source: Pediatr Rheumatol Online J. 2013 Dec 3;11:45. doi: 10.1186/1546-0096-11-45 (PMC4176131; doi:10.1186/1546-0096-11-45)

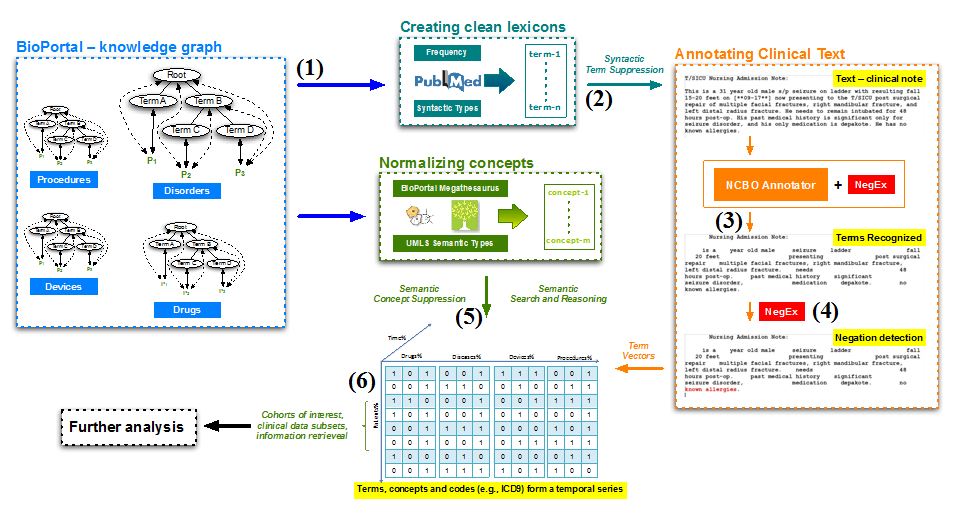

Supplement: Additional file 1: Figure S1 — Generation of the patient–feature matrix. This process (1) starts by downloading ~5.6 M strings for every term in ontologies from both UMLS and BioPortal as well as all trigger terms from NegEx and ConText, (2) uses term frequency and syntactic type information (e.g., predominant noun phrases) from MedLine to prune the set of strings into a clean lexicon, (3) applies the lexicon directly against the textual notes using exact string matching, (4) applies NegEx and ConText rules to identify negated terms and family history contexts respectively, (5) applies UMLS and BioPortal mappings and semantic type information to normalize terms into concepts that are grouped by drug, disease, device, or procedure, (6) and results finally in the patient–feature matrix. Each row of the matrix represents a single patient’s note and the timestamps of the notes induces a temporal ordering over the entire patient–feature matrix. [file 1546-0096-11-45-S1.jpeg]
